# Patient Journey and Unmet Needs in Hidradenitis Suppurativa: Insights from an Italian Survey

**DOI:** 10.3390/jcm15145735

**Published:** 2026-07-22

**Authors:** Vincenzo Bettoli, Alberto Maria Bertoldi, Massimo Donini, Natale Schettini, Eleonora Adamo, Lucia Casoli, Alice Messi, Arianna Tonelli, Diletta Valsecchi, Giuseppina Pintori

**Affiliations:** 1 Acne and Hidradenitis Suppurativa Clinic, O.U. Dermatology, Azienda Ospedaliero, University of Ferrara, 44124 Ferrara, Italy; natale.schettini@gmail.com; 2 Acne and Related Disorders Centre, 40125 Bologna, Italy; 3 Ospedale di Venezia e Mestre, 30122 Venezia, Italy; alberto.bertoldi@outlook.com (A.M.B.); massimo.donini@aulss3.veneto.it (M.D.); 4 Novartis Farma SpA, 20154 Milano, Italy; eleonora.adamo@novartis.com (E.A.); lucia.casoli@novartis.com (L.C.); alice.messi@novartis.com (A.M.); arianna.tonelli@novartis.com (A.T.); diletta.valsecchi@novartis.com (D.V.); 5 Passion People, 40100 Bologna, Italy; direzionepassionpeople@gmail.com

**Keywords:** hidradenitis suppurativa, quality of life, patient journey, biologic therapy, psychological impact

## Abstract

**Background/Objectives**: Hidradenitis suppurativa (HS), a chronic, inflammatory skin condition, severely affects quality of life. Despite advances in understanding its pathophysiology, major gaps persist in diagnosis and management. This study examined the journeys and unmet needs of Italian participants via an online survey, focusing on diagnostic delays, treatments, and quality of life for HS. **Methods**: Data were collected through a survey shared by the HS patient association in a dedicated Facebook group, using 45 closed-ended questions through Computer-Assisted Web Interviews. A total of 320 participants completed the survey in January 2023. **Results**: Participants reported consultations with approximately five clinicians before receiving a formal diagnosis, with a 10-year delay. Dissatisfaction with primary care was high (73%). Dermatologists played a central role in disease management. Only 24% of participants were on biologic therapy, despite the higher satisfaction levels compared to other treatments. HS was associated with quality-of-life impairments, particularly in psychological wellbeing (58%), daily functioning, and economic productivity. Unmet needs included better psychological support, improved access to biologics, nutritional guidance, and increased awareness of the disease among healthcare professionals. **Conclusions**: HS imposes considerable physical, psychological, and economic burdens. Earlier diagnosis, improved treatment strategies, and enhanced patient-centered care are essential to reducing this burden.

## 1. Introduction

Hidradenitis suppurativa (HS), also known as acne inversa, is a chronic, relapsing inflammatory skin disorder predominantly affecting apocrine-gland-bearing areas, such as the axillae, groin, and anogenital regions [1,2,3]. Characterized by painful nodules, abscesses, sinus tracts, and scarring, HS significantly impacts the quality of life (QoL) and often requires complex management strategies [4,5,6]. Indeed, beyond its physical manifestations, HS presents psychological and social burden. Chronic pain, recurrent flares, and visible lesions contribute to stigmatization and social withdrawal. Impairments in intimate relationships and sexual functioning are frequently reported, together with reduced self-esteem and altered body image. Anxiety, depression, and other psychiatric comorbidities are associated with this condition. These multidimensional consequences further complicate disease management and significantly affect patients’ overall wellbeing [3,7]. The Hurley Staging System is a common method for classifying HS, categorizing the disease into three stages of increasing severity, from single abscesses to extensive scarring [8,9]. Hidradenitis Suppurativa Severity Score System (IHS4) assesses the evolution of the disease [10].

The pathophysiology of HS involves genetic predispositions, immunological and hormonal dysregulation, and environmental triggers [4]. Immune dysregulation plays a pivotal role in the disease process, characterized by an overproduction of pro-inflammatory cytokines such as TNF-α and IL-17, leading to chronic inflammation and tissue damage [3,4,8,11]. Globally, HS prevalence is estimated from 0.0003% to 4.1%, with females exhibiting a 2–3 times higher risk compared to males [4,12]. In Italy, the prevalence is approximately 0.056% [2].

Risk factors for HS include a family history, reported for 30% of patients, as well as lifestyle habits like smoking and obesity, which can worsen inflammation and increase mechanical friction [3,8].

Despite increasing knowledge of HS pathophysiology, several gaps remain along the diagnostic and therapeutic pathway. Patients frequently experience prolonged delays before receiving a correct diagnosis, limited access to advanced treatments, insufficient psychological and nutritional support, and variable awareness of the disease among healthcare professionals [13,14]. These unmet needs involve broader aspects of patient-centered care. In addition, higher disease severity correlates with greater impairment in QoL and psychological distress, and likely with persistent unmet clinical needs among patients with moderate-to-severe HS [3,13].

While previous studies have utilized standardized, quantitative questionnaires to survey patients, there is a need for a deeper exploration of patients’ experiences. This study aims to address that gap by examining patients’ experiences with HS, including the impact of HS on their QoL, diagnostic and therapeutic journeys, and the most frequently used information channels.

## 2. Methods

### 2.1. Survey Methodology and Participants

The study conducted in 2023 by Elma Research included the following phases:

1. Targeted literature review to identify domains related to patient QoL and their diagnostic and therapeutic journeys.
2. Domains’ validation by the Italian patient association “Passion People APS” (PAG). This process ensured the identification of the final list of domains to be included in the survey (Supplementary Materials).
3. Questionnaire development with the following sections:
  - Study introductory section, presenting the overall context of the study.
  - Informed consent section, describing the aim and structure of the project and data management, according with local regulation.
  - Eligibility screening section, to determine the inclusion criteria (adults being affected by HS).
  - Personal data section, collecting socio-demographic and job-related variables to allow for sub- and stratified analyses, and to test for systematic differences in preferences based on these characteristics.
  - HS-specific sessions covering patient journey and HCPs consulted, therapy, impact of HS on QoL, sources of information consulted by respondents.
4. Questionnaire pilot phase: the questionnaire, comprising 45 closed-ended questions, was reviewed by the PAG to assess its cognitive burden and comprehensibility.
5. Questionnaire main phase: the questionnaire was digitized, and the link was shared through the Facebook group “Hidradenitis Suppurativa/HS”. Participants were asked to provide a written informed consent before starting the questionnaire. Only patients respecting the inclusion criteria could proceed and be interviewed for 15 min using the Computer-Assisted Web Interviews methodology.

### 2.2. Statistical Analysis

The data were analyzed between 27 November and 19 December 2023.

Descriptive statistics were used to summarize survey data. Categorical variables were reported as counts and percentages and compared between groups using chi-square tests. Fisher’s exact test was used when appropriate and to confirm the robustness of the results. Risk differences were estimated and 95% confidence intervals (CI) were calculated using the Newcombe method. Quantitative variables were compared between groups using PROC TTEST with the Satterthwaite methods; results are reported as mean differences with 95% CI. All tests were two-sided and a *p*-value < 0.05 was considered statistically significant. Considering the sample size and the absence of marked skewness in the analyzed variables, this approach was considered appropriate for group comparisons.

Analyses were performed using SAS 9.4 (TSLEVEL 1M8, STAT 15.3; SAS Institute Inc., Cary, NC, USA).

## 3. Results

### 3.1. Patient Characteristics

A total of 320 patients were interviewed. Overall, 40% of the participants were from Northern Italy, 38% from the South and Islands, and 22% from the Central regions.

Patients’ characteristics are available in Table 1. Responders were mainly women (82%). The average age was 40.8 (SD = 11.9) years, and 51% were ≤40 years. Forty-nine percent of respondents were married or cohabiting with a partner, and 27% were single.

Most patients had a high school education (57%), and 60% were employed. Among those employed (*n* = 193), 45% worked full-time, while 15% worked part-time. Seventy-three percent of patients reported at least one comorbidity, with the most common being psychological or psychiatric conditions (24%) and obesity (23%). Other comorbidities included gynecological problems (17%), autoimmune dermatological and rheumatological diseases, and other dermatological conditions (both 14%).

Disease severity was available for 284 patients, and 35% had severe, 33% moderate, and 21% had mild disease.

The severity level was determined by a physician for 71% of these cases, while for the remaining 29% it was based on self-assessment.

### 3.2. Journey to Diagnosis

**Figure 1:**
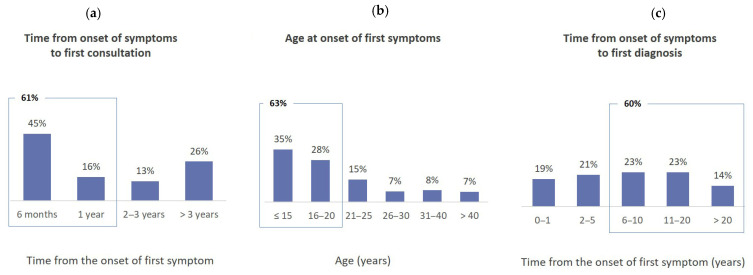
Time from the onset of symptoms to first consultation, age at first symptoms, and time from onset of symptoms to diagnosis: (**a**) *n* = 320; (**b**) *n* = 320, (**c**) *n* = 313.

The mean age at the onset of the first clinical features was 21 (SD = 10.7) years. Most of the 320 patients (61%) had their first consultation with a physician within one year after the clinical features appeared (Figure 1a). Overall, 313 patients received a diagnosis of HS, and 63% of those were ≤20 years at the onset of the first symptom(s) (Figure 1b). Within this group, a diagnosis was made between 6 and more than 20 years after the onset of the first clinical features for 60% (average of 10 years, SD = 9.2) (Figure 1c).

Among the 320 patients, 55% consulted between two and five clinicians, while 31% consulted more than six clinicians (average of 5.2, SD = 4.0). Dermatologists were the primary specialists providing the diagnosis in 198 (72%) out of 276 patients, mostly in private (56%) settings, and were the main reference for HS management (55%).

Participants followed by general practitioners reported higher dissatisfaction compared with those followed by dermatologists (73% vs. 40%, risk difference 32%, 95% CI 16.8–46.0; *p* < 0.001).

Overall, 79% of the 320 patients were dissatisfied with the ease of obtaining an HS diagnosis, with the majority being not at all satisfied.

### 3.3. Treatment and Support

**Figure 2:**
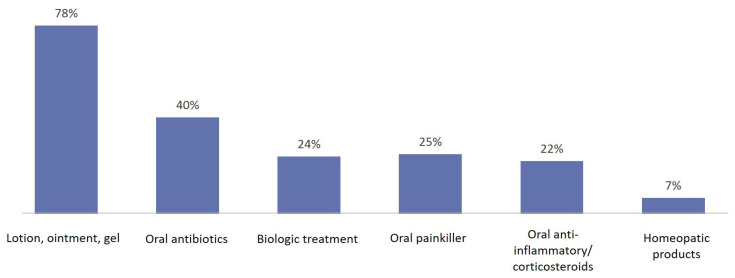
Current pharmacological treatment (*n* = 320).

Of the 320 patients, 78% were using topical treatments at the time of the survey. For systemic treatments, oral antibiotics were the most common (40%). Oral painkillers were reported by 25% of patients, biologics by 24%, followed by oral anti-inflammatory/corticosteroids (22%), and homeopathic products (7%) (Figure 2).

Compared with patients not receiving biologic therapy, patients treated with biologics more frequently reported concomitant autoimmune dermatological and rheumatological diseases (26% vs. 13%; risk difference 13.4%, 95% CI 3.9–24.7; *p* = 0.004) and autoimmune gastrointestinal diseases (13% vs. 5%; risk difference 7.9%, 95% CI 1.3–17.4; *p* = 0.014). They were also significantly more often followed at an HS reference centre (83% vs. 37%; risk difference 46.2%, 95% CI 34.8–54.8; *p* < 0.001) and more frequently reported a severe disease stage (58% vs. 33%; risk difference 25.9%, 95% CI 13.5–37.4; *p* < 0.001). No statistically significant differences were observed in the proportion of patients consulting a clinician within one year from symptom onset (70% vs. 60%; risk difference 10.1%, 95% CI −2.1 to 20.8; *p* = 0.103).

**Figure 3:**
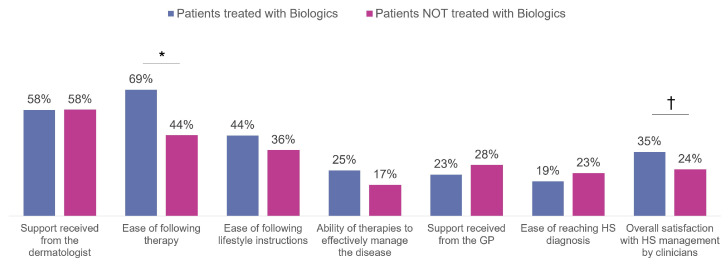
Patient-reported satisfaction according to biologic treatment status. Comparison of patient-reported satisfaction and support-related outcomes between patients treated with biologics and those not treated with biologics. (Patients treated with biologics, *n* = 77; patients not treated with biologics, *n* = 243); * *p* < 0.001; † *p* = 0.051.

Patients on biologic treatment were highly satisfied compared to those not treated with biologics. Statistically significant differences were noted in the ease of following therapy (69% [*n* = 53] vs. 44% [*n* = 107], risk difference 25.1%, 95% CI 12.7–35.9; *p* < 0.001). Overall satisfaction with HS management by clinicians showed a numerical increase among patients treated with biologics (35% [*n* = 27] vs. 24% [*n* = 58]), with borderline evidence for a between-group difference (risk difference 11.0%, 95% CI −0.02 to 23.0; *p* = 0.051) (Figure 3).

In addition to pharmacological treatments, 54% of 320 patients had undergone surgery, though laser therapy was less frequently used. Moreover, 78% of participants independently managed closed abscess dressings, 71% handled open abscess dressings, and 56% performed independent drainage of abscesses.

Beyond medical therapies, participants reported adopting several lifestyle precautions to mitigate the severity of their HS, such as wearing comfortable clothing (92%), following a proper diet (72%), avoiding alcohol consumption (71%), managing their body weight (70%), using specific detergents (69%), avoiding sun exposure (51%), engaging in physical activity (42%), and avoiding smoking (39%). Despite the declared attention to nutrition, only 46% of patients consulted a dietician/nutritionist, with 27% making an appointment on their own initiative.

### 3.4. Impact on QoL and Wellbeing

Most participants reported that HS had a significant impact on their QoL, with 48% indicating a high impact, 25% reporting a medium impact, and 27% experiencing a low impact. Among those who reported a high impact (*n* = 152), 43% were from the South and Islands. In addition, 30% had psychological or psychiatric comorbidities, and 18% had autoimmune dermatological and rheumatological diseases. Patients reporting a higher HS impact on QoL also reported a significantly longer time from symptom onset to diagnosis compared with those reporting a lower impact (mean difference 4.06 years, 95% CI 2.03–6.09; *p* < 0.001). In addition, patients with higher QoL impact more frequently reported a severe stage of HS (48% vs. 23%; risk difference 25.4%, 95% CI 15.0–35.1; *p* < 0.001). Patients with higher QoL impact also reported consulting a higher number of clinicians on average (5.8 vs. 4.6), although this comparison was descriptive.

On the Numeric Rating Scale (NRS) for pain, 66% of patients rated HS-related pain as moderate or higher (NRS score ≥ 7). Notably, 40% reported worst possible pain (NRS score 9–10), while only 13% indicating no or low pain (NRS score 0–3). Participants also identified fatigue (56%) and itching (47%) as the most impactful symptoms of HS, with a NRS score ≥ 7.

**Figure 4:**
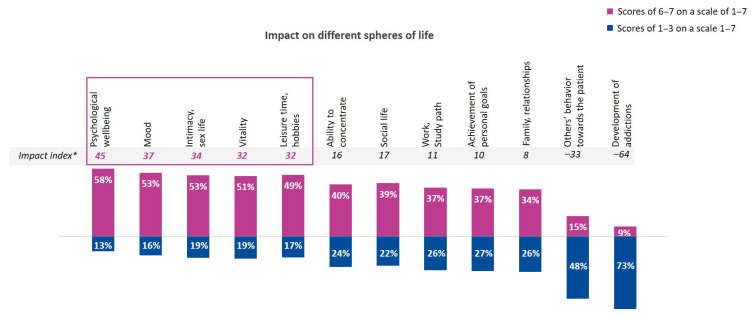
HS impact on different spheres of life (*n* = 320). * Percentage difference between high impact (scores 6–7 on a scale of 1 to 7) and low impact (scores 1–3 on a scale of 1 to 7).

HS had a substantial impact on personal wellbeing, with 58% of patients indicating high impairment in psychological wellbeing (Figure 4). Despite this, only 32% had sought support from a mental health professional, with 23% doing so on their own initiative. Mood, intimate/sex life, vitality, and leisure time/hobbies were also significantly affected, with participants indicating severe impacts in these areas (Figure 4). Other aspects of life, including the ability to concentrate, social life, work/study path, achievement of personal objectives, and family/relationships, were also adversely affected, although to a lesser degree, with impact indices ranging from 8 to 17 (Figure 4).

The negative impact extended to patients’ perceptions of others’ behavior toward them and the development of addictions, with notably low impact indices of −33 and −64, respectively (Figure 4). This suggests that HS affects not only the patients’ internal experiences but also their external environment and relationships.

### 3.5. Economic Impact

Less than half of the patients provided estimates of their HS-related expenses; 26% spent €0–500 annually on medical consultations. Additionally, 22% spent the same amount on medical services. Patients receiving biologic therapy reported lower average annual expenses for visits and medical services compared with those not treated with biologics, although these findings should be interpreted with caution, as this comparison was only descriptive.

HS negatively impacted the productivity for 74% of working participants, causing them to miss work (69%) and losing job opportunities (25%). Moreover, 20% were either mobbed or discriminated. Notably, 30% of respondents identified HS as the reason for giving up their job or working part-time (7%). Additionally, 33% of part-time workers indicated that HS influenced their decision to reduce their working hours.

### 3.6. Informative and Support Resources

The internet emerged as the primary source of information, with 68% consulting social networks, 57% relying on websites, and 52% seeking information from patient associations. These were key sources for 64% of patients. Medical professionals played a secondary role, with 39% consulting private dermatologists and even fewer relying on dermatologists at reference centers, public dermatologists, GPs, or other specialists. Specialists were seen as a key source of information only by 31% of patients.

Among the 182 patients relying on websites, 48% visited disease-specific sites, 47% consulted patient association websites, 37% used health-related sites, and 36% accessed forums and blogs. Online encyclopedias and specialist websites were also commonly used, while fewer patients consulted YouTube, hospital websites, or other specialized online services.

### 3.7. Unmet Needs

**Figure 5:**
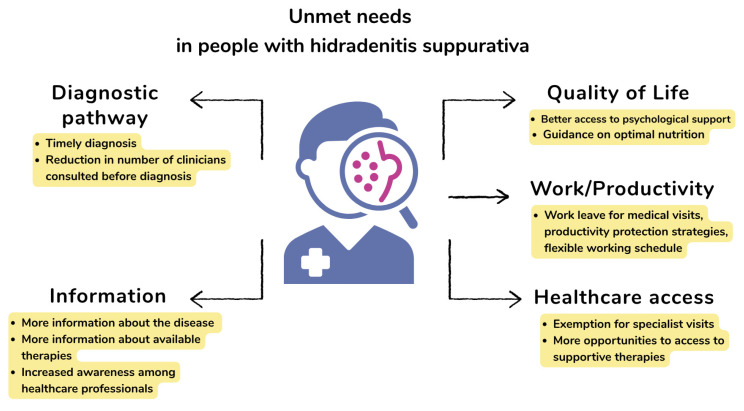
Key unmet needs identified in patients with HS.

One of the most pressing needs was obtaining an exemption for specialist visit (78% of patients). Other important needs included increased awareness among healthcare professionals (62%), better knowledge of the patient support services available (57%), and more information about available therapies (56%). Additional needs included receiving more information about the disease (50%), gaining more psychological support (46%), and obtaining guidance on the best nutrition practices (49%). Furthermore, 40% of patients expressed the need for work leave to attend medical visits. Social support was also a notable concern, with 37% seeking the chance to share their condition with patients facing the same challenges. Lastly, patients identified the need for greater access to supplementary therapies—such as meditation or yoga (32%)—more involvement in treatment decisions (30%), and additional information from or about patient associations (22%). A summary of the relevant unmet needs emerging from the study are reported in Figure 5.

## 4. Discussion

This survey provides a comprehensive overview into the experiences of HS patients in Italy, highlighting the significant clinical and socio-health challenge of HS, and its profound, multidimensional impact on diagnosis, treatment, and patients’ QoL. The data underscore the urgent need for better management to improve QoL of those affected.

### 4.1. Diagnostic Journey and Healthcare Interactions

One of the most striking findings is the lengthy delay in HS diagnosis, with participants waiting an average of 10 years from the onset of symptoms. Over half of the respondents reported waiting between 6 to 20 years for a diagnosis, a delay that is longer than the 7-year average reported in some studies [3,5,8], but consistent with other studies indicating delays of 7–10 years [13,15]. Consistent with previous literature [12,16], patients reporting a higher impact of HS on quality of life also reported longer diagnostic delays in our survey, supporting the relevance of timely diagnosis in reducing disease burden. This delay is associated with increased disease burden and impairment of QoL, contributing to dissatisfaction with medical care and reluctance to seek further treatment. Early identification of HS would allow prompt treatment that can minimize the risk of disease progression and associated comorbidities [3,13]. Notably, quality improvement initiatives, such as those by Boothby-Shoemaker and colleagues, have demonstrated that reducing appointment lead-time delays and improving clinical documentation can significantly enhance the overall care experience for HS patients [17].

### 4.2. Treatment and Disease Management

Dermatologists have an important role in reducing diagnosis delays and managing HS, being the primary point of contact for both initial diagnosis and ongoing management. Satisfaction with dermatologists was generally positive, while general practitioners were perceived as less supportive. This indicates a gap in primary care for HS patients, suggesting the need for improved training and resources for general practitioners to better support patients with this complex condition.

Despite the established benefits of biologic therapies for moderate to severe HS, only 24% of the participants were receiving such treatments. This percentage is consistent with trends reported in other studies [6,14,18]. Patients receiving biologic therapy reported higher levels of satisfaction with their treatment pathway and its management, as compared with patients not receiving biologics.

Many participants adopted lifestyle changes, like wearing comfortable clothing and managing weight, but the limited use of dieticians and specialized services suggests they often manage their condition independently. This is concerning given that obesity was the second most common comorbid condition in this survey.

### 4.3. Economic, Social and QoL Impact

HS impacts patients’ QoL, with nearly half of participants reporting a high impact on their daily lives. Psychological wellbeing is significantly affected. However, despite these challenges, only 32% sought support from a mental health professional, pointing to a significant unmet need for psychological care. The high levels of pain, fatigue, and itching, combined with the impact on mood, intimate relationships, and overall vitality, further illustrate the comprehensive toll that HS takes on patients’ lives. Moreover, the burden associated with HS may be further amplified by the presence of comorbidities, including depression, anxiety, obesity, metabolic disorders, and immune-mediated diseases such as inflammatory bowel disease, whose coexistence has been associated with increased disease severity and management complexity [19,20].

Economically, HS imposes a substantial burden, with costs primarily for medications and healthcare visits. Patients receiving biologic therapy reported lower expenses for medical visits and services compared with those not treated with biologics; however, this observation should be interpreted cautiously given the descriptive nature of the analysis.

The study also reveals a significant impact on employment, with 74% of working participants reporting decreased productivity and 30% attributing job loss or resignation to HS. This aligns with findings from other studies indicating that HS patients often have lower socioeconomic status and higher unemployment rates compared to the general population [14,21,22].

### 4.4. Informative Resources and Unmet Needs

The study reveals a reliance on non-professional sources of information, such as social networks and patient associations, rather than healthcare professionals. While these sources provide accessible information, the low reliance on medical professionals suggests a potential gap in the communication and support provided by clinicians. This highlights the need for better informational outreach by healthcare providers, particularly dermatologists and HS specialists, to ensure that patients receive accurate and comprehensive guidance.

Participants identified numerous areas where current healthcare services are lacking, including the need for increased awareness among healthcare professionals, more psychological support, nutritional guidance, and improved access to biologic therapies. Similar unmet needs have been identified in other studies, where patients emphasized the need for better medical management and supportive care [1].

### 4.5. Limitations

The participants were active in an online support group, likely having greater awareness of HS and its management, which may not reflect the broader HS patient population, introducing potential bias. Additionally, data were self-reported and may be subjected to recall bias and inaccuracies. Although the sample reflects the Italian population and aligns demographically with North American and European cohorts, it may not capture the full diversity of experiences across socioeconomic and geographic groups, potentially limiting the generalizability of the results [12,14]. Another limitation is the lack of Information regarding the scoring system used to determine disease severity. Although clinicians confirmed the severity reported by patients, the specific score employed for this assessment was not disclosed in the survey. Additionally, pain evaluation was limited to the day of the survey, which may not accurately reflect the typical fluctuations associated with HS.

### 4.6. Recommendations for Future Research and Practice

Based on the descriptive findings of this survey and in line with existing evidence, to adequately improve HS management and address patients’ needs, a coordinated intervention is essential. Although further studies exploring patient perspectives in different contexts to improve the generalizability of the findings are needed, a possible intervention to ameliorate HS management should include: (i) timely diagnosis and continuous training for healthcare professionals; (ii) improved access to advanced therapies, ensuring equitable availability of biologic treatments for all HS patients, regardless of location or socioeconomic status; (iii) multidisciplinary, patient-centered care, integrating psychological and nutritional support to address specific comorbidities associated with HS; (iv) implementation of strategies to bridge the information gap between patients and healthcare providers, promoting two-way communication and shared knowledge.

National guidelines and Diagnostic and Therapeutic Care Pathways should be developed and implemented, based on the latest scientific evidence and tailored to the specific health profiles of patients.

An integrated, patient-centered strategy is essential to improve the HS care pathway, alleviate the disease burden, and align care with the latest standards of assessment and protection. Such measures are vital to enhancing patients’ well-being and ensuring a fair, efficient, and sustainable healthcare system.

## 5. Conclusions

This survey of patients in Italy highlights the burden of HS, both in terms of physical symptoms and impact on psychological, social, and economic wellbeing. There is urgent need for earlier diagnosis, improved access to effective treatments, multidisciplinary and personalized approach, better education and support for both patients and healthcare providers, and increased attention to the psychological and social dimensions of HS. Addressing these needs will require a coordinated effort from dermatologists, primary care providers, mental health professionals, and policymakers.

## Figures and Tables

**Table 1:** Patient characteristics (*n* = 320).

Demographics	*n*	%
*Age*		
Up to 30 years	75	23
31–40 years	91	28
41–55 years	103	32
Over 55 years	51	16
*Gender*		
Female	263	82
Male	56	18
Unknown	1	^§^
*Marital status*		
Married/cohabiting	156	49
Single	87	27
In a relationship	49	15
Separated/divorced	23	7
Widower	5	2
*Level of education*		
Graduation and beyond	80	25
High school	181	57
Secondary school	56	18
Elementary school	1	- ^§^
No formal education	2	1
*Working status*		
Working	193	60
Full-time workers	144	45
Part-time workers	49	15
Not working	104	33
Unemployed	41	13
Student	19	6
Housewife	33	10
Retired	11	3
Other	23	7
*Comorbidities*		
At least one comorbidity	234	73
Psychological/psychiatric problems	77	24
Obesity	75	23
Gynecological problems	55	17
Autoimmune dermatological and rheumatological diseases	44	14
Other dermatologic diseases (Fox Fordyce, pyoderma…)	43	14
Hypertension	42	13
Joint diseases	39	12
Diabetes/metabolic hormonal dysfunction	38	12
Immune system dysfunction	21	7
Autoimmune gastrointestinal diseases	19	6
Heart diseases	10	3
Cancer	4	1
No comorbidities	86	27
*Current stage of the disease*		
(Hurley stage III)	111	35
(Hurley stage II)	106	33
(Hurley stage I)	67	21
Unknown	36	11

^§^ Values <1%.

## Data Availability

The authors confirm that the data supporting the findings of this study are available within the article.

## Supplementary Materials

The following supporting information can be downloaded at: https://www.mdpi.com/article/10.3390/jcm15145735/s1. SUPPLEMENTAL SURVEY.

## Abbreviations

The following abbreviations are used in this manuscript:

HS	Hidradenitis suppurativa
IHS4	Hidradenitis Suppurativa Severity Score System
NRS	Numeric Rating Scale
PAG	Passion People APS
QoL	Quality of life
