# Supplementary material for: Patient Journey and Unmet Needs in Hidradenitis Suppurativa: Insights from an Italian Survey
Source: J Clin Med. 2026 Jul 22;15(14):5735. doi: 10.3390/jcm15145735 (PMC13412834; doi:10.3390/jcm15145735)
Supplement: Supplementary file 1 [file jcm-15-05735-s001.zip › jcm-4415762-supplementary.pdf]

SUPPLEMENTAL SURVEY

Survey full text

DESCRIPTIVE DATA

S1 Let's talk about Hidradenitis Suppurativa (HS). You…

1. Suffer from it personally
2. Other

S2 Age: |__|__|

S3 Sex information:

1. Male
2. Female
3. Other gender (non-binary)
4. Prefer not to answer

S4 Educational level

1. No formal education
2. Elementary school diploma
3. Middle school diploma
4. High school diploma
5. University degree or higher

S5 Marital status

1. Single
2. In a relationship
3. Married/cohabiting
4. Separated/divorced
5. Widowed

R1 In which region do you live? (List for choice)

S6 You live…

1. Alone
2. With someone (parents, partner, children)

S7 Occupation

1. Full-time worker
2. Part-time worker
3. Retired
4. Unemployed

5. Homemaker
6. Student
7. Other

S8 Besides Hidradenitis Suppurativa (HS), do you currently suffer from any of the following conditions?

1 Diabetes/metabolic or hormonal disorders
2 Immune system disorders
3 HIV
4 Hypertension
5 Joint diseases (arthropathy, arthritis, etc.)
6 Autoimmune dermatological and rheumatological diseases (Lupus/SLE, psoriasis, psoriatic arthritis, etc.)
7 Autoimmune gastrointestinal diseases (e.g. Crohn's disease, ulcerative colitis, etc.)
8 Heart Diseases
9 Obesity
10 Gynecological issues (e.g. polycistic ovary syndrom, etc.)
11 Psychological/psychiatric conditions (e.g. depression, anxiety etc.)
12 Cancer
13 Other dermatological issues (acne, folliculitis, pityriasis, Fox-Fordyce disease, pyoderma gangrenosum, etc.)
14 Other
15 None of these

DIAGNOSTIC PATHWAY AND REFERENCE DOCTORS

M1 Approximately how old were you when the first symptoms of Hidradenitis Suppurativa (HS) appeared?

|__|__| years

M2 How long after the first symptoms did you consult a doctor for the first time regarding this issue?

1. After about 6 months
2. After about 1 year
3. After about 2-3 years
4. After more than 3 years

M3 And how old were you when you received the first diagnosis of Hidradenitis Suppurativa?

|__|__| years

M4 How many different doctors did you consult over time before receiving the HS diagnosis?

|__|__| doctors

M5 Besides you, has any other family member ever suffered from HS?

1. Yes → Who?? 1. Father/2. Mother/3. Brother - Sister/4. Other relative
2. No
3. I don't know

M6 Which doctors have you consulted over time for your HS problem?

General Practitioner (GP)

Hospital Dermatologist (co-pay required)

Public Health Service (ASL) Dermatologist (co-pay required)

Private Dermatologist (in clinic or hospital, full cost paid by patient)

General Surgeon

Plastic/Cosmetic Surgeon

Gynecologist

Proctologist

Andrologist

Pediatrician

Endocrinologist

Immunologist

Infectious Disease Specialist

Rheumatologist

Other

M7 Which of these is your primary reference or managing HS?

General Practitioner (GP)

Dermatologist

Hospital Dermatologist (co-pay required)

Public Health Service (ASL) Dermatologist (co-pay required)

Private Dermatologist (in clinic or hospital, full cost paid by patient)

Surgeon

General Surgeon

Plastic/Cosmetic Surgeon

Gynecologist

Proctologist

Andrologist

Pediatrician

Other

M8 Which doctor was the first to diagnose HS?

General Practitioner (GP)

Dermatologist

Hospital Dermatologist (co-pay required)

Public Health Service (ASL) Dermatologist (co-pay required)

Private Dermatologist (in clinic or hospital, full cost paid by patient)

Surgeon

General Surgeon

Plastic/Cosmetic Surgeon

Gynecologist
Proctologist
Andrologist
Pediatrician
Other

M9 Are you currently being treated at a specialized HS center?

1. Yes
2. No
3. I don’t know

M10 Have you ever consulted any of the following professionals for HS-related issues?

a. Dietitian/Nutritionist

1. Yes, on the advice of a dermatologist
2. Yes, on the advice of a general practitioner
3. Yes, on my own initiative
4. No

b. Psychiatrist/Psychologist/Psychotherapist

1 Yes, on the advice of a dermatologist
2 Yes, on the advice of a general practitioner
3 Yes, on my own initiative
4 No

M11 What is the current severity stage of your HS?

1. MILD
2. MODERATE
3. SEVERE
4. I don’t know

M12 Was the severity level communicated by a doctor, or is it your own assessment?

1. A doctor told me
2. Personal assessment

M13 Now, try to assess the current severity level of your HS by choosing one of the images below:

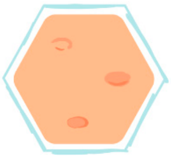	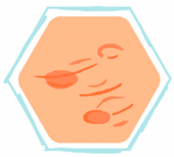	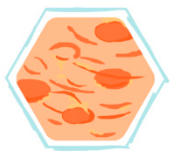

Single or sporadic abscesses, usually without tunnel or fistula formation. The affected area is limited to a small skin region.	Recurring or multiple abscesses, often with the formation of subcutaneous tunnels or fistulas. The affected area is larger and involves multiple body regions.	Extensive and multiple abscesses, with numerous fistulas and subcutaneous tunnels. The affected area is very large and may affect many parts of the body.

4 I don't know

THERAPY

T1 What pharmacological treatment are you currently following for HS? antibiotici (terapia orale)

1. Antibiotics (oral therapy)
2. Anti-inflammatory drugs/corticosteroids (oral therapy)
3. Painkillers (oral therapy)
4. Biologic drug- Injectable therapy administered in the hospital
5. Biologic drug- Injectable therapy self-admistered at home
6. Cream, ointments, gels
7. Homeophatic products

T2 Who first prescribed the biologic drug?

1. Dermatologist
2. Rheumatologist
3. Gastroenterologist
4. Other

T3 How long after the diagnosis did the biologic therapy start?

1. Less than a year after diagnosis
2. About one year after diagnosis
3. About 2–3 years after diagnosis
4. About 4–5 years after diagnosis
5. More than 5 years after diagnosis

T4 Have you ever taken a biologic drug (injectable therapy administered in the hospital) for HS in the past?

1. Yes
2. No

T5 Have you ever undergone any of the following procedures for HS…?

Surgical intervention

Laser therapy performed by a dermatologist
Laser therapy performed by a medical specialist other than a dermatologist
Laser treatment performed by a non-medical operator

T6 How many times?

	n. of times
1. Surgical intervention	_____
2. Laser therapy performed by a dermatologist	_____
3. Laser therapy performed by a specialist other than a dermatologist	_____
4. Laser treatment performed by a non-medical operator	_____

T7 Besides medical treatments, certain lifestyles adjustments can help manage HS. Do you follow this behaviors?

	YES	*NO*
1. Following a healthy/proper diet		
2. Controlling body weight		
3. Avoiding smoke		
4. Avoiding alcohol consumption		
5. Avoiding sun exposure		
6. Engaging in physical activity		
7. Wearing comfortable clothing		
8. Using specific cleansers		

T8 Let's talk about wound care.

For dressing closed abscesses, do you seek medical assistance or do it indipendently?

1. Dressings done only by a doctor/nurse/healthcare professional
2. Dressings done only indipendently (by themselves or with the help of a family member/relative/friend)
3. Both methods

T9 And for dressing open abscesses, do you seek medical assistance or do it independently?

1. Dressings done only by a doctor/nurse/healthcare professional
2. Dressings done only indipendently (by themselves or with the help of a family member/relative/friend)
3. Both methods

T10 Regarding abscess drainage, do you seek medical assistance or do it independently?

1. Drenage done only by a doctor/nurse/healthcare professional
2. Drenage done only independently (by themselves or with the help of a family member/relative/friend)
3. Both methods

T11 Now, we'd like you to estimate the costs you incur due to HS.

On average, how much do you spend in a year on HS-related medical visits and healthcare services?

Medical visits |__|__|__|__|__| €/YEAR I don't know

Healthcare services |__|__|__|__|__| €/YEAR I don't know

T12 On average, how much do you spend per month on medications, treatments, and wound care for HS?

Medications (anti-inflammatories, painkillers, etc.) |__|__|__|__|__| €/MONTH I don't know

Ointments, creams, gels |__|__|__|__|__| €/MONTH I don't know

Supplements, natural/homeopathic remedies |__|__|__|__|__| €/MONTH I don't know

Disinfectants, gauze, bandages |__|__|__|__|__| €/MONTH I don't know

PATHWAY

P1 Indicate how satisfied you are with each phase of the treatment pathway by selecting the corresponding response

	Not at all satisfied	*Slightly satisfied*	*Fairly satisfied*	*Very satisfied*
1. Ease of obtaining an HS diagnosis				
2. Effectiveness of treatment in managing the disease				
3. Ease of following the prescribed treatment				
4. Ease of adhering to lifestyle recommendations				
5. Support received from the general practitioner				
6. Support received from the dermatologist				
7. Overall satisfaction with medical management of HS				

IMPACT ON THE QUALITY OF LIFE

Q1 How much does HS impact your life? Please use a scale from 1 to 7, where 1= no impact and 7= very high impact

No impact 😀 😀 🙂 😐 🙁 ☹️ ☹️ *Very high impact*

Q2 In what ways has HS affected your daily life? Please indicate the response that best describes your experience in the LAST MONTH

**In the last month, how much has your HS…**	*Not at all*						*Very much*
Limited your social life	1	2	3	4	5	6	7
Created relationship problems with your family/partner/close relatives	1	2	3	4	5	6	7
Caused intimacy/sexual problems	1	2	3	4	5	6	7
Negatively influenced how others behave towards you	1	2	3	4	5	6	7
Interfered negatively with your work/study progress	1	2	3	4	5	6	7
Negatively affected your psychological well-being	1	2	3	4	5	6	7
Interfered with your leisure activities/hobbies	1	2	3	4	5	6	7
Led you to develop dependencies (alcohol/substances)	1	2	3	4	5	6	7
Negatively affected your mood	1	2	3	4	5	6	7
Reduced your vitality	1	2	3	4	5	6	7
Interfered with your ability to concentrate	1	2	3	4	5	6	7
Compromised the achievement of your personal goals	1	2	3	4	5	6	7

Q3 How would you describe the symptoms caused by HS today? Please use a scale from 0 to 10, where 0= none, 1=minimal and 10=maximum

	none	MIN									MAX
Pain	0	1	2	3	4	5	6	7	8	9	10
Itching	0	1	2	3	4	5	6	7	8	9	10
Fatigue	0	1	2	3	4	5	6	7	8	9	10

Q4 Due to HS (directly or indirectly), have you ever experienced the following?

	Never	*Rarely*	*Sometimes*	*Often*	*Very often*
1. Psychological violence					

2. Physical violence					
3. Difficulties maintaining relationships with others					
4. Difficulties maintaining romantic relationships					
5. Discrimination					

If worker COD 1-2 A S7

Q5 Let's talk about the impact of HS on work activity. Due to HS (directly or indirectly), how often have you experienced the following at work?

	Never	*Rarely*	*Sometimes*	*Often*	*Very often*
1. Missed workdays					
2. Reduced productivity (lower concentration, fatigue, difficulty completing tasks, etc.)					
3. Workplace bullying (mobbing) or discrimination					
4. Missed career opportunities, promotions, or advancements					
5. Had to resign from a job		1 sì		2 No	
6. Being fired		1 sì		2 No	

Q6 Is your part-time employement related (directly or indirectly) to HS?

1. No, my part-time work has nothing to do with HS
2. Yes, I chose to work fewer hours due to the condition
3. Yes, my employer reduced my working hours due to my condition

Q7 Is your current unemployement related (directly or indirectly) to HS?

1. No, my unemployement is unrelated to HS
2. Yes, I chose to stop working due to the condition
3. Yes, my employer dismissed me due to my condition

INFORMATION

I1 How well-informed do you feel about Hidradenitis Suppurativa? Please respond using a scale from 1 to 7, where 1= not informed at all and 7= very well-informed.

Not informed at all 😞 😞 😟 😐 🙂 🙂 😊 *Very well-informed*

I2 Through which channels have you received the information you currently have about HS?

Medical channels

General Practitioner
Public dermatologist
Dermatologist from the referral center
Private dermatologist
Medical specialist other than a dermatologist

Family/friends
Digital channels

Patient associations / Non-profit foundations (Passion People, Inversa Onlus, …)
Social networks (e.g., Facebook group...)
Websites
WhatsApp groups

Pharmacist
Radio
School / work
Press (newspapers, magazines)
TV
Other (Please specify)

I2a What has been your primary source of information?

Medical channels

General Practitioner
Public dermatologist
Dermatologist from the referral center
Private dermatologist
Medical specialist other than a dermatologist

Family/friends
Digital channels

Patient associations / Non-profit foundations (Passion People, Inversa Onlus, …)
Social networks (e.g., Facebook group...)
Websites
WhatsApp groups

Pharmacist
Radio
School / work
Press (newspapers, magazines)
TV
Other (Please specify)

I3 Specifically, which websites have you used to find the information you have about HS?

1. Online encyclopedias (e.g. Wikipedia, etc.)
2. Websites dedicated to this specific condition
3. Pharmaceutical company websites
4. Institutional websites (Ministry of Health, local health authorities, etc.)

5. Patient association or non-profit organization websites
6. Specialist doctors' websites
7. Hospital/medical center/reference center websites
8. Health information websites
9. News websites
10. Youtube
11. Forums and blogs
12. Online services
13. Other

I4 What do you feel you need in order to better manage your HS? You may select multiple answers

1. Receive more explanations/information about the disease
2. Receive more explanations/information about available treatment
3. Be more involved in treatment decisions
4. Receive more psychological support
5. Have the opportunity to connect with other patients with the same condition
6. Obtain work leave to attend medical visits
7. Receive exemptions for medical visits
8. Greater sensitivity from healthcare professionals
9. Receive more information from/about patient associations
10. Learn more about available patient support services
11. Receive more information about the appropriate diet to follow
12. More references to integrated therapies (e.g. meditation techniques, yoga therapy, etc.)
13. Other
